# A data-driven Markov process for infectious disease transmission

**DOI:** 10.1371/journal.pone.0289897

**Published:** 2023-08-10

**Authors:** Chengliang Wang, Sohaib Mustafa

**Affiliations:** College of Economics and Management, Beijing University of Technology, Beijing, China; Universidad Nacional de Colombia, COLOMBIA

## Abstract

The 2019 coronavirus pandemic exudes public health and socio-economic burden globally, raising an unprecedented concern for infectious diseases. Thus, describing the infectious disease transmission process to design effective intervention measures and restrict its spread is a critical scientific issue. We propose a level-dependent Markov model with infinite state space to characterize viral disorders like COVID-19. The levels and states in this model represent the stages of outbreak development and the possible number of infectious disease patients. The transfer of states between levels reflects the explosive transmission process of infectious disease. A simulation method with heterogeneous infection is proposed to solve the model rapidly. After that, simulation experiments were conducted using MATLAB according to the reported data on COVID-19 published by Johns Hopkins. Comparing the simulation results with the actual situation shows that our proposed model can well capture the transmission dynamics of infectious diseases with and without imposed interventions and evaluate the effectiveness of intervention strategies. Further, the influence of model parameters on transmission dynamics is analyzed, which helps to develop reasonable intervention strategies. The proposed approach extends the theoretical study of mathematical modeling of infectious diseases and contributes to developing models that can describe an infinite number of infected persons.

## 1. Introduction

The Coronavirus Disease 2019 (COVID-19) has rapidly stretched across the globe since its discovery. The outbreak of COVID-19 caused serious damage to the lives and properties of the people, interrupted normal productive life, and caused great losses [1–3]. Globally, as of January 9 2023, more than 660 million cases of COVID-19 have been confirmed, which includes more than 6.6 million cases of death [4]. Until now, humans still do not have a good means to effectively fight the virus. The study [5] pointed out that COVID-19 will still be around us for a long time. Therefore, there is an urgent need to find effective methods to capture and predict the transmission process of COVID-19. In addition, the method can be applied to analyze the role and effectiveness of non-drug intervention strategies. Further, more economical and effective strategies for non-drug interventions may be developed based on the analyses.

The COVID-19 transmission process can be considered as containing multiple stages [6, 7]. The stages have different transmission rates but are highly infectious [8, 9]. The initial stages of the transmission process show an exponential increase [10–12]. A large percentage of COVID-19 patients are found to have more severe symptoms [2]. The process of transmission of COVID-19 also seems to be seasonally related [13, 14]. When winter temperatures are extremely low, the transmission of COVID-19 becomes very rapid [15]. However, the simple characteristics of the COVID-19 transmission process are already known. However, it is still important to continue studying the COVID-19 transmission process to closely forecast its development over time. At the same time, there are no drugs or ways to treat COVID-19 rapidly and effectively. Therefore, in the fight against COVID-19, non-drug intervention strategies continue to be used mainly to limit the process of COVID-19 transmission. Hence, after further understanding the process of COVID-19, on the basis of the characteristics of the transmission process, there is a need to develop effective strategies for control and prevention to limit economic losses.

So far, control measures against COVID-19 have attracted much attention, and studies have shown their effectiveness [16–19], which can be divided into two main ways: (1) early identification of cases [20–27]. (2) increase the distance of transmission of the virus, including travel restrictions [28–30], individual and family isolation [19, 20, 31, 32], increased social distance [33–36], and exposure restrictions [37–41]. Moreover, some scholars have studied the effectiveness of other nondrug intervention strategies. Sarkar et al. [42] and Khajanchi et al. [43] demonstrated that quarantining susceptible individuals and contact tracing can control the COVID-19 outbreaks. Kumar Rai et al. [44] and Sarkar et al. [45] studied the effect of environmental contamination on COVID-19 pandemic dynamics considering and not considering vaccination coverage, respectively. Also, Kumar Rai et al. [46] and Khajanchi et al. [47] found that encouraging hospitalization and quarantine through the media helped control the disease’s prevalence.

Since the onset of the COVID-19 epidemic, several mathematical modeling approaches have been used to assess and predict the transmission process of COVID-19. Some scholars study the early diffusion of COVID-19 based on the Poisson processes [48] and the Markov Chain Monte Carlo method [49]. The statistical method based on time series data seems effective [50–53], but it can only follow previous patterns with no ability to predict the changing trend. Modeling based on agent-based simulation techniques, i.e., computing by copying the pattern of individuals (agents) in transmitting the infectious disease, is also a reasonable approach [54–58]. However, this model relies on population-level parameters that are difficult to obtain, such as movement rate, distance, and virus infectivity parameters. Models based on AI techniques are also interesting and effective approaches [59–64], but their effectiveness may be questioned in the absence of sufficient training data set as there are many learning steps they rely on.

Scholars mostly evaluate and predict COVID-19 based on the susceptibility-infection-recovery (SIR) model originally proposed by Kermack and McKendrick in 1927 [65]. This model and its evolved variants have been used by many scholars to simulate or predict the short-term dynamics of infectious diseases [66–70], to assess the impact of intervention strategies [71–74], to evaluate the impact of vaccines [75, 76], and to assess the stability of infectious disease transmission [77, 78]. Similarly, some studies use differential equation methods [79, 80]. Further, to study the random factors in the process, some scholars studied the model combining the Markov process and SIR [81–83] and the model combining the Hidden Markov process with SIR [84–86]. A common feature of these studies is to consider the same state (Susceptible, Infective, or Removal) as a whole during the state transition process. It is easy to see that studying all individuals as a whole will yield different results than studying each individual individually. On the other hand, it is necessary to set a maximum value for the number of susceptible patients in the SIR model. However, the number of susceptible in a region is not constant if inter-regional travel is considered. This setting is not realistic.

Our work is motivated by the fact that existing models used to describe infectious diseases or predict the process of their transmission generally assume that regions are closed and ignore population movements. However, cross-regional movement of populations is very frequent in today’s world, and the impact of population movement can increasingly not be ignored in transmitting and controlling infectious diseases. When population movements are considered, the number of people who can be infected by an infectious disease (or the total population) in an area can be considered variable or infinite. In this paper, we aim to develop a method that allows for the close description and analysis of the complex transmission process of infectious diseases without setting the maximum number of susceptible individuals. The method develops a new infectious diseases Markov model based on the level-dependent Markov process with infinite state space. The model can accurately describe the main characteristics of the infectious disease transmission process by four basic parameters that can be estimated from public data [4]. Based on the proposed infectious diseases Markov model, we propose a simulation method with a heterogeneous structure to obtain the model’s results. Experiments on six COVID-19 cases have shown that the method can well capture the real increasing process of infectious disease-infected patients.

The main contributions of this study are:

1. In the context of globalization, it is very common for people to travel between different countries and regions. We consider this reality and propose a level-dependent Markov process model with an infinite state space to capture the transmission dynamics of infectious diseases. Unlike the SIR model, the model does not require a predetermined maximum number of susceptible individuals or populations. The proposed approach provides new ideas for research on modeling infectious diseases in the new globalized context.
2. The mathematical formula for solving the model is given, but the infinite matrix needs to be truncated during the solution. It is difficult to truncate the infinite matrix within the allowed error range. In order to perform the solution quickly, we developed a heterogeneous simulation technique to reflect the situations in which different batch transmissions co-exist.
3. Several numerical experiments with COVID-19 are given to verify the validity and suitability of the model proposed in this paper. The model proposed in this paper can effectively describe the process of infectious disease transmission that is stable or with turning points. And the error in applying the model to predict the infectious disease transmission process will also be smaller than that of existing models.
4. We also evaluated the effects of the key parameters on the transmission process over time. The obtained results allow us to evaluate the implemented non-drug intervention strategies and provide the theoretical basis for the design of appropriate non-drug intervention strategies.

## 2. Materials and method

This section describes the methods adopted in this study. Section 2.1 describes the setting of the research problem and data. Section 2.2 gives the key parameters for constructing the model and how to estimate the parameters. Section 2.3 gives a Markov model with an infinite state space and a mathematical solution to this model, after which a heterogeneous simulation technique is proposed.

### 2.1. Research setting and data

We developed a new infectious disease Markov model to describe and characterize the diffusion of large-scale epidemics of infectious diseases, using COVID-19 as an example. The model is characterized by level dependence and the inclusion of infinite state space. This model divides the infectious diseases transmission process into two parts: Ⅰ. the generation process of new cases in phase form; Ⅱ. The disappearing process of cases is to cure, self-heal, or die one by one. Assuming that new cases can be detected in a timely manner, the emergence of new cases to their cure, self-healing, or death can be regarded as a service process. Under this assumption, we can view the transmission process of infectious diseases as a level-dependent quasi-birth and death (QBD) process with infinite service desks.

In the following part of simulation verification, we take COVID-19 as a typical example of an infectious disease and simulate different regions of the world that may be of concern: the state of New York, India, Egypt, South Korea, Italy, and Mexico. In selecting the data period, we chose 20 days of data within November 2020 for these countries because we hope our results can reflect the performance of COVID-19 transmission under more than one condition. The 20-day data results from the combined effects of climate, population density, seasonality, and different mitigation measures in different countries. At the end of our study, when we focus on the impact of different containment and mitigation strategies, we assume that some mitigation strategies change the value of transmission parameters to some extent and study the contribution of different parameters to the transmission through this method.

### 2.2. Parameters and measures

This section simplifies the random factors in the infectious disease transmission process into four key parameters. Based on Markov process theory, the parameters are assumed to obey exponential distribution [13]. The four key parameters can be described specifically:

1. *λ*: The rate of infection. The rate of transmission of one batch per infectious disease patient. The two infection batches are assumed to follow an exponential distribution in their interval.
2. *μ*: The rate of disappearing. The rate of disappearance of infectious diseases patients who recover themselves, die or are cured. Note that the time spent by patients disappearing also follows an exponential distribution.
3. *d*: The batch size of infection. The number of patients included in a batch infected by infectious diseases patients.
4. *k*: The starting number of actively infected cases. At the start of the observational period, the number of patients who are suffering from this infectious disease in the observation area.

By using some actual data, we can use the following method to define the following basic parameters: *k*, *λ*, *d*, and *μ*. Assume that there is a total of *m* observed epidemic data. And *N*_*i*_ is the total number of active cases reported on day *i*, *n*_*i*_ is the number of newly infected patients increased on day *i* with comparison to day *i*-1, and *c*_*i*_ is the number of newly disappeared infected patients on day *i* with comparison to day *i*-1.

The starting number of the actively infected cases (*k*) can be obtained directly from the actual statistics without calculation. Then, the parameters *λ* and *d* are given in Eq 1.

$$\lambda d=\frac{\frac{n_{1}}{N_{1}}+\frac{n_{2}}{N_{2}}+\frac{n_{3}}{N_{3}}+\cdots +\frac{n_{m-1}}{N_{m-1}}+\frac{n_{m}}{N_{m}}}{m},$$ (1)

where depending on the actual data, *d* maybe 0, 1, 2, 3,… Similarly, Eq 2 gives the calculation method for parameter *μ*.


$$\mu =\frac{\frac{c_{1}}{N_{1}}+\frac{c_{2}}{N_{2}}+\frac{c_{3}}{N_{3}}+\cdots +\frac{c_{m-1}}{N_{m-1}}+\frac{c_{m}}{N_{m}}}{m}.$$ (2)

The values of these four parameters (*λ*, *μ*, *d*, and *k*) can be determined by the approach described above. The number of active cases who are suffering from this infectious disease in each period is our most concerned and critical indicator during an epidemic. Therefore, we need to develop a model that uses these four parameters to forecast the increasing process of the active case number.

### 2.3. Model and computer experiment procedure

**Fig 1:**
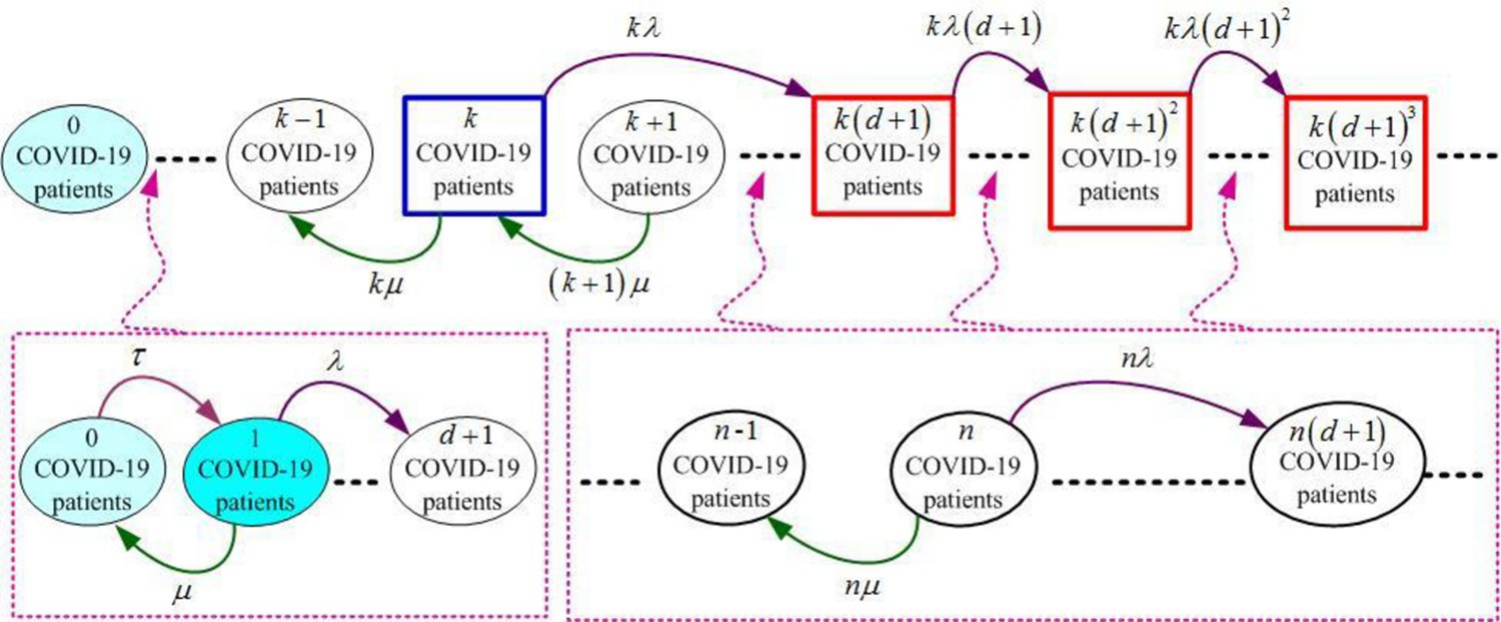
A graph of the state transfer relationship of the model.

An infectious disease Markov model is developed to forecast the increasing process of the active case number. In the model, let the state at the time *t* be *E*[*N*(*t*)], where the state represents the number of actively infected cases. And according to the infectious disease transmission process, the relationship of the transition between the states of the model ({*N*(*t*):*t*≥0}) is given in Fig 1.

where *τ* represents the rate of emergence of the first infected person in an area. It is usually small and does not affect the transmission of infection when it is created.

From Fig 1, it is possible to divide all states into different level sets, defined as follows:

level0:{1,2,3,…,k−1} (3)

level1:{k,k+1,k+2,…,k(d+1)−1} (4)

$$\mathrm{level}\;2:\;\{k(d+1),k(d+1)+1,k(d+1)+2,\ldots ,k{\left(d+1\right)}^{2}-1\}$$ (5)

$$\mathrm{level}\;n:\;\{k{\left(d+1\right)}^{n-1},k{\left(d+1\right)}^{n-1}+1,k{\left(d+1\right)}^{n-1}+2,\ldots ,k{\left(d+1\right)}^{n}-1\},n\geq 3$$ (6)

By using these level sets (level *n*, *n* = 0,1,2,…), the infinitesimal generator of this infectious disease Markov model ({*N*(*t*):*t*≥0}) is given by

$$Q=(\begin{array}{ccccc} A_{0,0} & A_{0,1} & & & \\ A_{1,0} & A_{1,1} & A_{1,2} & & \\ & A_{2,1} & A_{2,2} & A_{2,3} & \\ & & \ddots & \ddots & \ddots \end{array})$$ (7)

where, *A*_*i*,*j*_ is the rate matrix of the transfer from level *i* to level *j*. *A*_*i*,*i*_ is the square matrix with the same dimension as level *i*. The dimensions of the other *A*_*i*,*j*_ can be determined based on *A*_*i*,*i*_. This details of *A*_*i*,*j*_ is presented in the Supporting information (S1 File). Let the initial probability vector **ω** of this Markov model be

ω=(0,0,…,0,1,0,0,…) (8)

where the (*k*+1)th cell of **ω** is 1, indicating that the probability of the initial state being *k* is 1. Suppose *p*_*n*_(*t*) is the probability that the Markov model is in state *n* at time *t*, i.e.


$$p_{n}(t)=P\{N(t)=n\},\;n=0,1,2,\ldots$$ (9)

Then, the transient probability vector (**P**(*t*)) of the Markov model at time *t* can be written as:

$$P(t)=(p_{0}(t),p_{1}(t),p_{2}(t),p_{3}(t),\ldots )$$ (10)

According to the Chapman-Kolmogorow equation, **P**(*t*) can be obtained from Eq (11).


$$P(t)=\omega \times e^{Qt},\;t\geq 0,$$ (11)

Based on the transient probability vector at time *t*, the average number of active cases at time *t* can be easily obtained by Eq 12.


$$E[N(t)]=P(t)\times {\left(0,1,2,3,\ldots \right)}^{T},\;t\geq 0.$$ (12)

It is easy to see that *Q* is an infinite matrix. During the solution process, it is necessary to truncate this infinite matrix and keep the error within the allowed range. It is difficult to truncate the matrix *Q*. For this reason, we developed a heterogeneous computer simulation technique based on this infectious disease transmission model. In the simulation process, the coexistence of different batch sizes of infection was considered, that is, the overall transmission process could be regarded as the union of the infection transmission processes of different batch sizes. The weights of the transmission processes with different infection batches in the combination are different.

**Fig 2:**
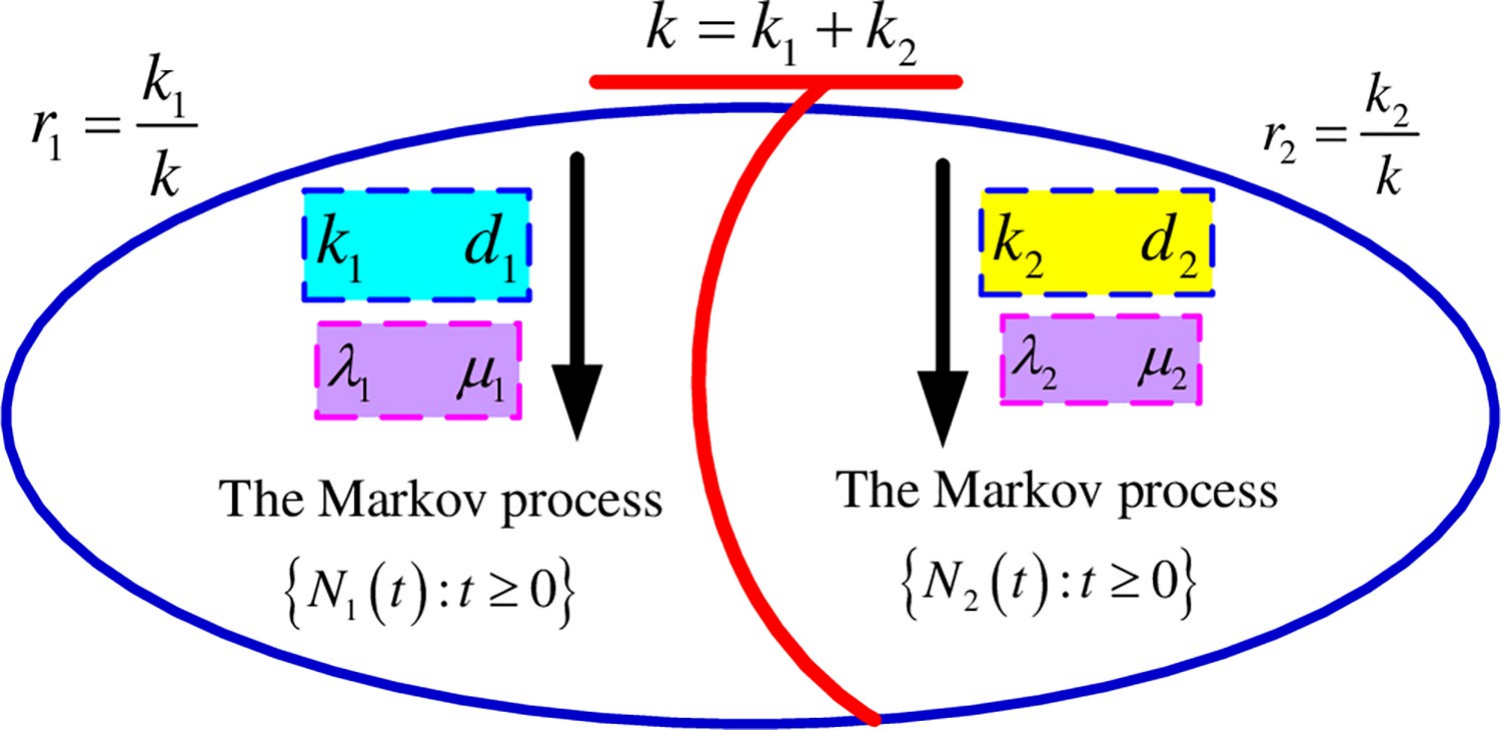
The Markov model with two groups of infectious batches.

The infectious disease transmission process in the coexistence of two infection batches is given in Fig 2. In Fig 2, *k* is the total number of actively infected cases for all groups at the start of the observation, *k*_*i*_ is the starting number of the actively infected cases in the group *i*, *r*_*i*_ is the weight value of the group *i*, *λ*_*i*_ is the rate of infection in group *i*, *μ*_*i*_ is the rate of disappearing in group *i*, and *d*_*i*_ is the batch size of infected patients in group *i*. The weight value for a given group *i* is the ratio between the number of actively infected cases in the group *i* and the total number of actively infected cases at the beginning of the observation period, i.e. ri=kik. The total infected batch size is obtained by weighting the average infected batch sizes of the two groups, i.e. $\bar{d}=r_{1}d_{1}+r_{2}d_{2}$.

Note that

$$E[N(t)]=E[N_{1}(t)]+E[N_{2}(t)]$$ (13)

We adjusted the value of weight *r*_*i*_ for the groups of different batches in the simulation experiment to make them nearly to the real reported values.

## 3. Results

In this section, using COVID-19 as an example, we describe the transmission of COVID-19 in some regions by applying the methods proposed in this paper and show how to evaluate the effects of interventions. Section 3.1 describes the process of simulating increasing activity cases of COVID-19 using actual data and compares the simulation results with the actual situation. In section 3.2, we experimentally illustrate the effect of each parameter on the COVID-19 transmission process over time.

### 3.1. Simulation results

In this subsection, COVID-19 was used as a typical example of infectious disease, and the increasing process of COVID-19 activity cases in 6 regions during the 20-day observation period was simulated to reflect the transmission of COVID-19. The key parameters in these examples were obtained by the approach in section 2.2 and based on actual data [4]. Then, the increasing process of COVID-19 active cases in these examples was simulated based on the infectious disease Markov model and heterogeneous simulation techniques proposed in this paper and compared with the real situation.

New York, India, and Egypt were simulated first. The simulation results for New York are presented in the main text, and the simulation results for India and Egypt are presented in the Supporting information (S1 and S2 Tables, S1 and S2 Figs). They represent the transmission process without turning points. Their transmission parameters did not change significantly during the observation period. This is because no intervention strategy was adopted, or the intervention strategy did not significantly change the transmission process of the pandemic during this period.

**Fig 3:**
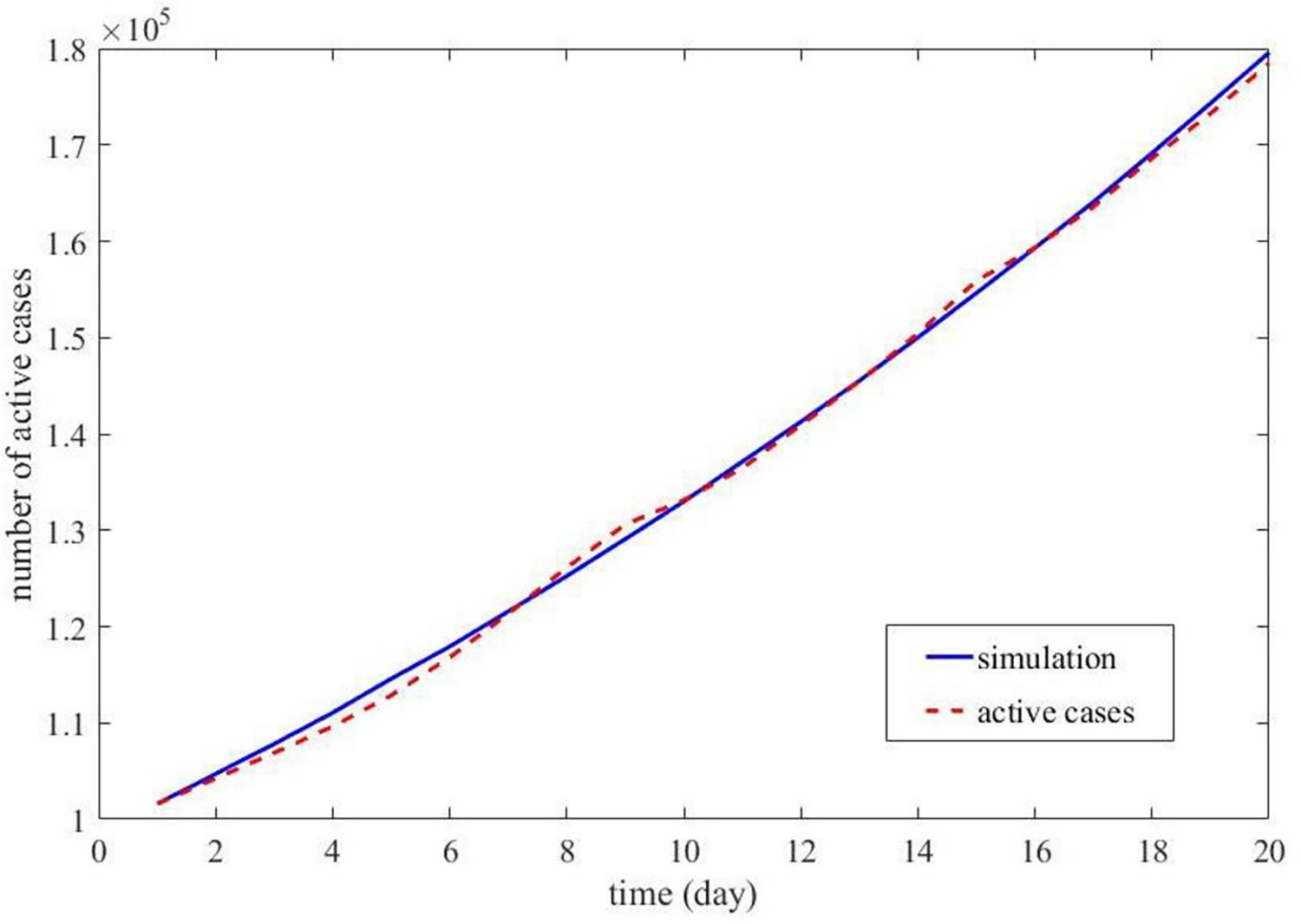
The comparison of simulation results with reported data in New York State.

The epidemic situation in the United States has aroused a lot of attention, and New York is a state of concern in the United States, so first, the epidemic in New York State was simulated. Based on the COVID-19 data reported for New York State, as given in Table 1, the following parameters for the New York State epidemic could be determined. *k* = 101592, and according to Eqs (1) and (2), the mean infection rate (*λ*) and disappearing rate (*μ*) were given as 0.035073852 and 0.006084398, respectively. Then, the increasing number of COVID-19 activity cases in New York State was simulated. The batches of infection were determined to be *d*_1_ = 1 and *d*_2_ = 2, and the two groups’ infection processes with infectious batches of 1 and 2 had weights (*r*) of 0.971 and 0.029, respectively. A comparison of the results of the simulation with those of the reported real data is shown in Fig 3.

**Table 1:** Reported COVID-19 data for New York State for November 6–25.

Date	Accumulated Confirmed Patients	Newly Infected Patients Daily	Disappearing Patients	New Disappearing Patients Daily	Active Cases
6Nov	559161	3241	457569	721	101592
7-Nov	562577	3416	458342	773	104235
8-Nov	565929	3352	459035	693	106894
9-Nov	569508	3579	459873	838	109635
10-Nov	573465	3957	460601	728	112864
11-Nov	578076	4611	461267	666	116809
12-Nov	583133	5057	461817	550	121316
13-Nov	588605	5472	462469	652	126136
14-Nov	593767	5162	463161	692	130606
15-Nov	597394	3627	464257	1096	133137
16-Nov	601457	4063	465006	749	136451
17-Nov	606624	5167	465711	705	140913
18-Nov	611988	5364	466524	813	145464
19-Nov	617741	5753	467298	774	150443
20-Nov	623242	5501	467354	56	155888
21-Nov	628808	5566	469471	2117	159337
22-Nov	634035	5227	470455	984	163580
23-Nov	640356	6321	471737	1282	168619
24-Nov	645834	5478	472576	839	173258
25-Nov	651830	5996	473349	773	178481

Fig 3 depicts the comparison of the simulation results obtained using our method with the actual COVID-19 transmission process in New York State. The blue solid line represents the simulation results, and the red dashed line represents the actual number of active cases in the Fig 3. The comparison shows that the simulation results of the COVID-19 transmission process obtained according to our proposed method are roughly the same as the actual COVID-19 transmission process using the parameters derived from the actual data.

To further validate the proposed model, we also simulated the epidemic in India and Egypt from November 1 to 20, 2020. The data used for the simulations and the results obtained are shown in the Supporting information (S1 and S2 Tables, S1 and S2 Figs). This shows that the proposed model enables to catch the COVID-19 transmission dynamics well when there is little fluctuation in the transmission parameters.

Unlike the three examples above, the transmission parameters may be turning due to the interventions or the disasters encountered. Therefore, the epidemic in Korea, Italy, and Mexico was further simulated, and there is a turning point (*t*_*c*_) in the growth curve of the activity cases for these three examples. Similar examples with turning points can be found in the literature [16]. The existence of these turning points reflects that the transmission process of COVID-19 in these examples was affected by the intervention strategy or other reasons. Therefore, we also included a turning point (*t*_*c*_) in the simulation of the processes in these three regions.

**Fig 4:**
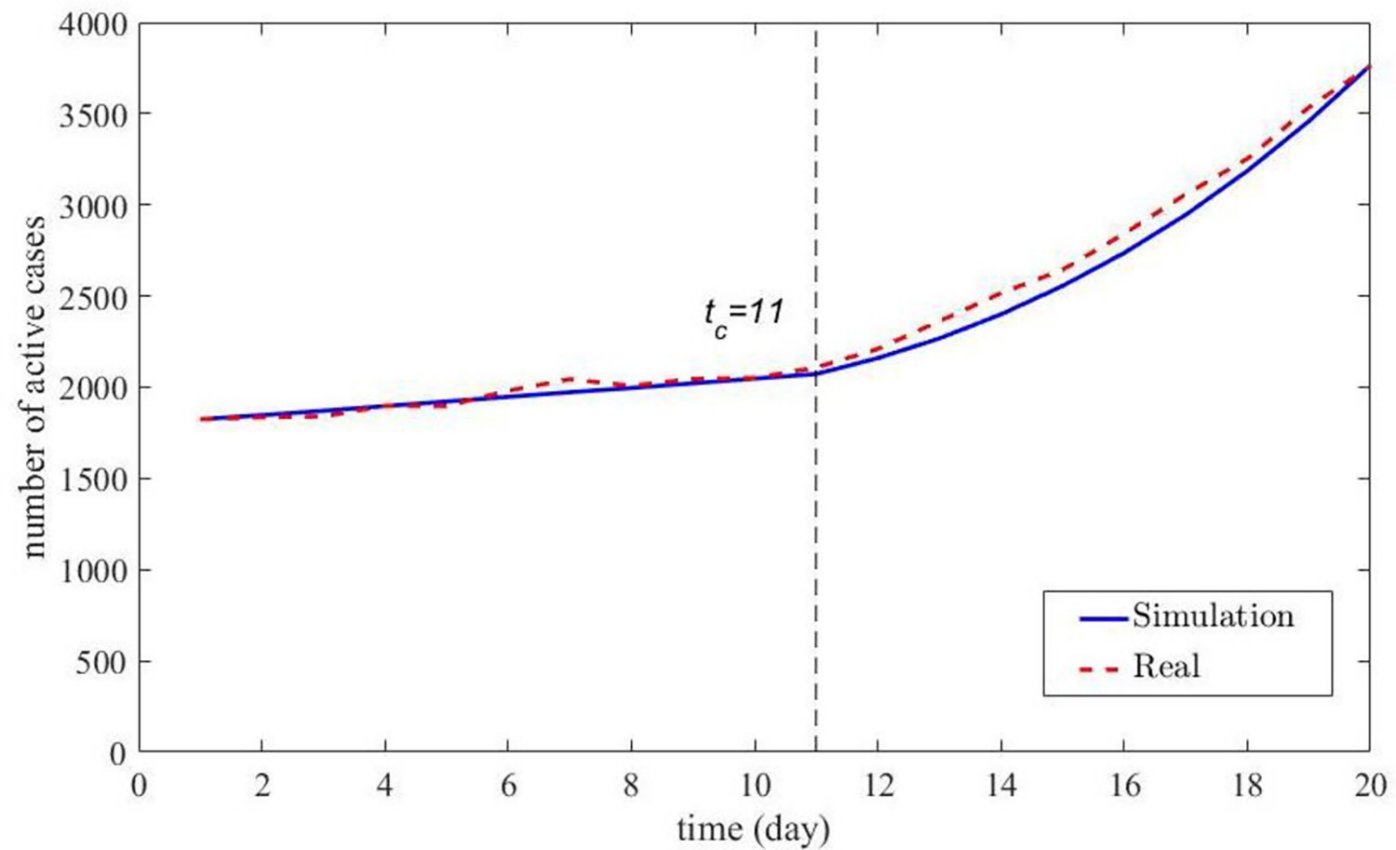
The comparison of simulation results with reported data in South Korea.

First, based on the reported data related to COVID-19 in Korea from November 2 to 21, 2020, given in Table 2, a turning point (*t*_*c*_) was found in the COVID-19 transmission process. After calculation, it can be obtained that *k* = 1825, the average infection rate (*λ*) and disappearing rate (*μ*) before the turning point, *t*_*c*_, are 0.062135947 and 0.053096363, respectively. Their values equal 0.09774732 and 0.038994525, respectively, after the turning point, *t*_*c*_. In the simulation, the infection batches were determined to be *d*_1_ = 1 and *d*_2_ = 2. Before the turning point *t*_*c*_, the weights (*r*) of the two groups equal 0.940 and 0.060, respectively. And after the turning point *t*_*c*_, they become 0.434 and 0.566, respectively. The simulation results are compared with the reported data in Fig 4.

**Table 2:** Data on COVID-19 for South Korea was reported from the 2nd to the 21st of November.

Date	Accumulated Confirmed Patients	Newly Infected Patients Daily	Disappearing Patients	New Disappearing Patients Daily	Infected Patients
2-Nov	26807	75	24982	119	1825
3-Nov	26925	118	25090	108	1835
4-Nov	27050	125	25210	120	1840
5-Nov	27195	145	25297	87	1898
6-Nov	27284	89	25387	90	1897
7-Nov	27427	143	25446	59	1981
8-Nov	27553	126	25509	63	2044
9-Nov	27653	100	25645	136	2008
10-Nov	27799	146	25753	108	2046
11-Nov	27942	143	25891	138	2051
12-Nov	28133	191	26025	134	2108
13-Nov	28338	205	26128	103	2210
14-Nov	28546	208	26184	56	2362
15-Nov	28769	223	26253	69	2516
16-Nov	28998	229	26354	101	2644
17-Nov	29311	313	26469	115	2842
18-Nov	29654	343	26596	127	3058
19-Nov	30017	363	26764	168	3253
20-Nov	30403	386	26868	104	3535
21-Nov	30733	330	26971	103	3762

Fig 4 shows the comparison of the simulation results obtained using our method with the actual transmission process of COVID-19 in South Korea. The blue solid line in Fig 4 indicates the simulation results, and the red dashed line indicates the number of active cases and *t*_*c*_ indicates the turning points in the transmission process. The comparison results show that for the COVID-19 transmission process with a turning point, the simulation results obtained according to our proposed method are approximately the same as the actual data. In this case, the simulation results were obtained using the parameters calculated based on the actual data.

Similarly, to validate that the proposed model can track the dynamics of transmission processes with turning points, the COVID-19 transmission processes in Italy and Mexico were also simulated. The data used for the simulations and the results obtained are shown in the Supporting information (S3 and S4 Tables, S3 and S4 Figs). This indicates that the proposed model can also track the transmission dynamics of the COVID-19 transmission process with a turning point.

According to all the previous simulation results, the following Table 3 is obtained ($\bar{d}=(d_{1}r_{1}+d_{2}r_{2})$).

**Table 3:** Key parameters in the dissemination of COVID-19 in each country.

Country		State of New York	India	Egypt	South Korea	Italy	Mexico
	*k*	101592	561908	1903	1825	613358	158429
*t*<*t*_*c*_	*λ*	0.035073852	0.088146484	0.085434063	0.062135947	0.049487386	0.027329732
	*μ*	0.006084398	0.101284201	0.045978143	0.053096363	0.023181119	0.030691817
	$\bar{d}$	1.029	0.999	1.054	1.060	1.001	0.980
*t*≥*t*_*c*_	*λ*	0.035073852	0.088146484	0.085434063	0.09774732	0.030904889	0.051140063
	*μ*	0.006084398	0.101284201	0.045978143	0.038994525	0.031409968	0.034605204
	$\bar{d}$	1.029	0.999	1.054	1.566	0.450	1.500

### 3.2. Influence of parameters

In the following, we assess how three parameters that non-drug intervention strategies may influence impact the infectious disease transmission process over time. The three parameters include *k*, *λ*, and *d*. Note that non-drug interventions can only limit the transmission of the virus and have little impact on patients who are already ill. Therefore, it is assumed that the non-drug intervention strategies that can be taken at present cannot affect *μ*.

To assess the effect of the parameters, we introduced a new indicator $\rho_{\theta ,\tilde{\theta }}$ and defined

$$\rho_{\theta ,\tilde{\theta }}=\frac{E[N_{\theta }(t)]}{E[N_{\tilde{\theta }}(t)]},$$ (14)

where, *θ* and $\tilde{\theta }$ denote the value of each parameter before and after changing the nondrug intervention strategy, respectively; and *E*[*N*_*i*_(*t*)] denotes the average number of infected patients with parameter value *i*.

**Fig 5:**
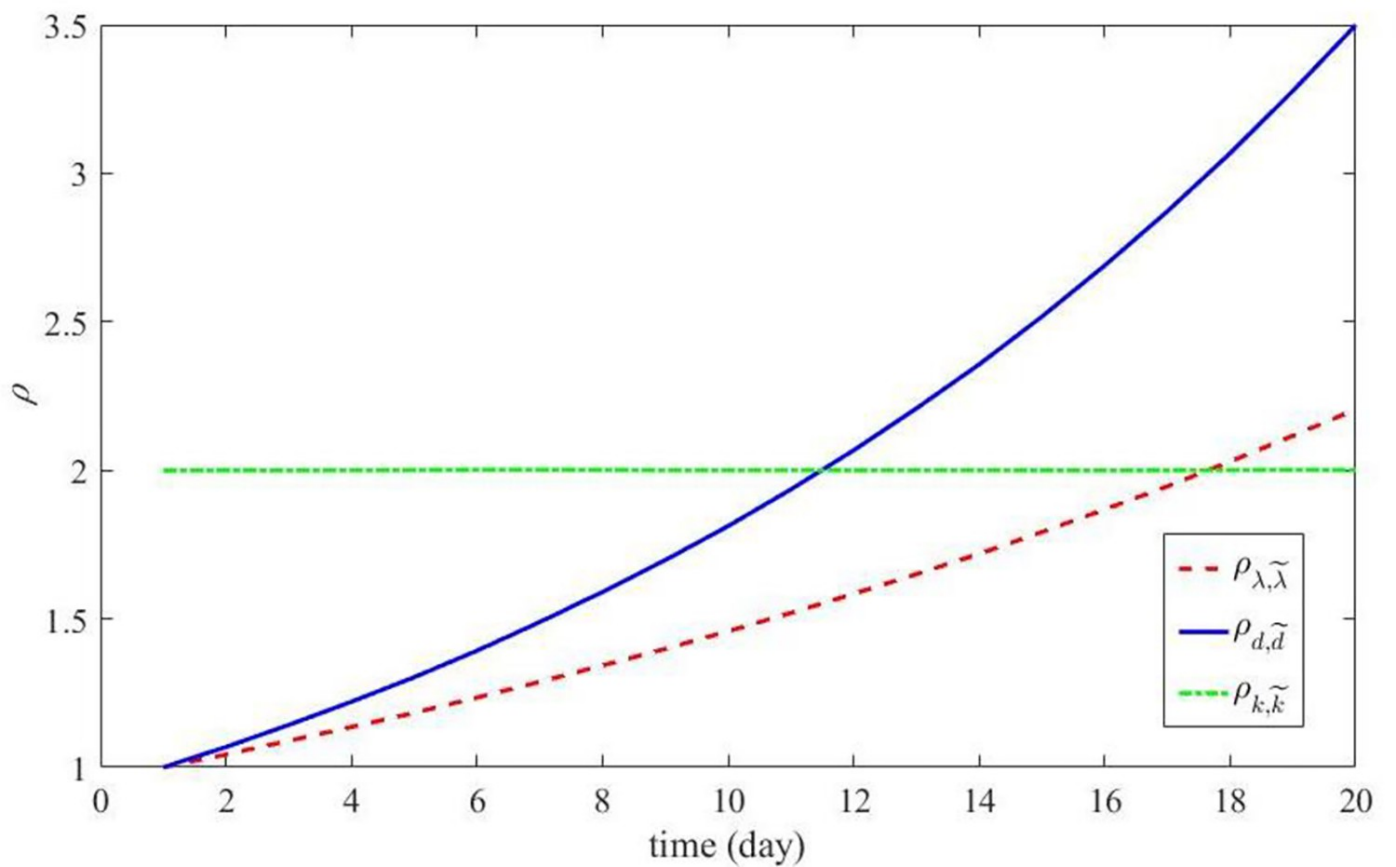
The trend of *ρ* in Egypt with time when changing *k*, *λ*, and *d*.

After that, we first use Egypt as an example and analyze the role of each parameter using Egyptian data. The COVID-19 transmission parameters for Egypt were as follows: *k* = 1903; *λ* = 0.085434063; *μ* = 0.045978143; and two sets of weights (*r*) of 0.946 and 0.054 for *d*_1_ = 1 and *d*_2_ = 2, respectively. After that, we assumed that each parameter was changed by the same proportion to half of its original value due to non-drug interventions: $\tilde{\lambda }$ = $\frac{1}{2}\lambda$; $\tilde{k}$ = $\left\lceil \frac{1}{2}k\right\rceil$. In order to make $\tilde{\bar{d}}$ = $\frac{1}{2}\bar{d}$, let *d*_1_ = 1 is changed to ${\tilde{d}}_{1}$ = 0, *d*_2_ = 2 is changed to ${\tilde{d}}_{2}$ = 1; ${\tilde{r}}_{1}$ = 0.518, ${\tilde{r}}_{2}$ = 0.482 are changed from *r*_1_ = 0.946, *r*_2_ = 0.054, respectively. The trend of *ρ* with time after changing the parameters is given for Egypt in Fig 5.

Fig 5 shows the effects of changing *k*, *λ*, and *d* on the COVID-19 transmission process in Egypt. The green, blue, and red lines in Fig 5 represent the trend of the effects of changing *k*, *λ*, and *d* over time, respectively. The results show that change *k* reduces the number of active COVID-19 cases and the effect does not change over time. Changing *λ* and *d* have a small effect on the number of active COVID-19 cases in the early days, and this effect increases significantly over time.

**Fig 6:**
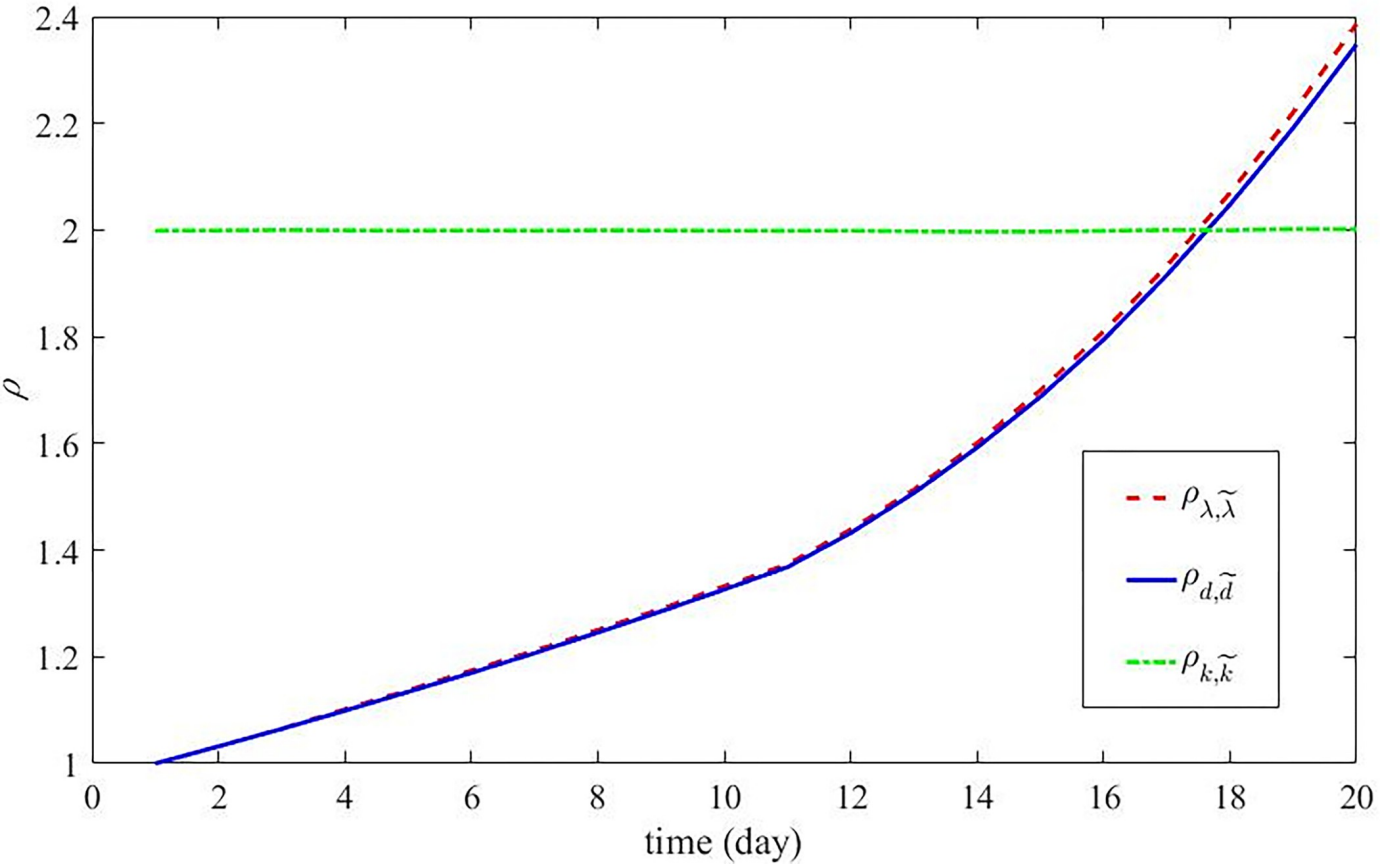
The trend of *ρ* in South Korea with time when changing *k*, *λ*, and *d*.

Further, taking South Korea as an example, we analyze the role of each parameter again when there is a turning point in the transmission process. The COVID-19 transmission parameters for South Korea were as follows: *t*_*c*_ = 11; *k* = 1825; *λ* before and after *t*_*c*_ are 0.062135947 and 0.09774732, respectively; *μ* before and after *t*_*c*_ are 0.053096363 and 0.038994525, respectively; the weight of group $d_{1}^{1}$ = 1 is 0.940 before *t*_*c*_; that of group $d_{2}^{1}$ = 2 is 0.060 before *t*_*c*_; the weight of group $d_{1}^{2}$ = 1 is 0.434 after *t*_*c*_; and the weight of group $d_{2}^{2}$ = 2 is 0.566 after *t*_*c*_. Similar to the previous example, the parameter becomes half of the original one. $\tilde{\lambda }$ = $\frac{1}{2}\lambda$; $\tilde{k}$ = $\left\lceil \frac{1}{2}k\right\rceil$; $d_{1}^{i}$ = 1 is changed to ${\tilde{d}}_{1}^{i}$ = 0, $d_{2}^{i}$ = 2 is changed to ${\tilde{d}}_{2}^{i}$ = 1 for *i* = 1, 2; ${\tilde{r}}_{1}^{1}$ = 0.470, ${\tilde{r}}_{2}^{1}$ = 0.530, ${\tilde{r}}_{1}^{2}$ = 0.217, ${\tilde{r}}_{2}^{2}$ = 0.783 are changed from $r_{1}^{1}$ = 0.940, $r_{2}^{1}$ = 0.060, $r_{1}^{2}$ = 0.434, $r_{2}^{2}$ = 0.566, respectively. The trend of *ρ* with time after changing the parameters is given for Egypt in Fig 6. The effects of the three parameters in Korea were found to be similar to those in Egypt.

Fig 6 depicts the effects of changing *k*, *λ*, and *d* on the transmission process of COVID-19 in South Korea. The green, blue, and red lines in Fig 6 represent the trends of the effects of changing *k*, *λ*, and *d* over time, respectively. The results obtained are the same as those in Fig 5. Changing *k* decreases the number of active COVID-19 cases and the effect does not change over time. Changing *λ* and *d* have a small effect on the number of active COVID-19 cases in the early days, and this effect increases significantly over time.

## 4. Discussion

In Figs 3 and 4 in the main text and Figures in the Supporting information (S1–S4 Figs), it can be seen that there is a good match between the simulation results output based on the proposed method in this paper and the actual data, regardless of whether there are turning points in the transmission process of COVID-19. Figs 5 and 6 shows the role of the model’s key parameters (*λ*, *d*, and *k*) for the model. How a non-drug intervention strategy affects parameter values can be derived by calculating and analyzing parameter values before and after the application of the intervention strategy. The influence of non-drug intervention strategies on the growth process of patients with COVID-19 infection and the transmission process of COVID-19 over time was evaluated using parameters as a medium. In addition, the future increase process of patients infected with COVID-19 can be predicted by extending the length of time of the simulation. Further, the results can be applied to purposefully adjust or develop measures that can effectively control COVID-19 to reduce economic losses due to measure implementation.

From the simulation of stable regions (Fig 3, S1 and S2 Figs) in the observation period: The four key parameters of the proposed Markov model for COVID-19 transmission can be calculated from the actual data [4] (Table 1, S1 and S2 Tables). These parameters comprehensively reflect the complex transmission situation in the observation area, including population density, climate, seasonality, etc. The actual spread process of COVID-19 is a stochastic and complex process, and the patients who are already infected with COVID-19 may infect new patients with different infectious batches. Because the infectious batches in our proposed model can only be integers, it is difficult to characterize the problem of the COVID-19 transmission process by just one infectious disease Markov process. To effectively investigate this problem, it is possible to consider the overall COVID-19 transmission process as the combination of multiple groups that are all independent COVID-19 transmission models. Combining multiple groups of COVID-19 transmission models can capture the dynamics of the increasing number of patients with COVID-19 infection within a region. According to our experimental findings, the increasing dynamics of the number of patients with COVID-19 infection in a region can be effectively captured by considering at least two different groups of COVID-19 transmission models. The heterogeneity of patient transmission does not affect the application of the model.

According to the simulation of regions with turning points (Fig 4, S3 and S4 Figs), we link the turning points with human intervention measures and natural environment changes. This study identifies the turning points in the observation period for several examples through actual data (Table 2, S3 and S4 Tables). By comparing the values of the parameters before and after the turning point, the control effect of the intervention strategy can be assessed. Note that there is a delay period between the time of implementation of the non-drug intervention strategy and the time of the turning point. A particular intervention strategy usually adopted will impact multiple parameters in the COVID-19 transmission process.

We identify the possible causes of turning points by finding out what measures the country took before the turning point. We suppose this change may be due to the relaxation of the social distance restriction in Korea on November 1, 2020. After a few days of transmission, this led to the uncontrollable transmission of the epidemic in the winter. The curfew policy and emergency imposed in some Italian regions after November 5, 2020, could be the reason for the turning point *t*_*c*_ = 12 in Italy. On November 11, 2020, Mexico was hit by a hurricane. The reduction of social distance between people caused by centralized placement may be the cause of *t*_*c*_ = 7 in Mexico. In this study, the focus is not to prove which measure causes the turning points but to better describe the COVID-19 transmission process through turning points. So, it is acceptable to find the cause of turning points this way. If the reader is interested, the location of the turning point can be found through simple outlier detection and other methods.

At the same time, the parameter changes around the turning point reveal that: (1) The implementation of curfews and emergency state strategies in parts of Italy was effective in controlling the increase in the number of infected patients and the transmission of COVID-19. This indicates that the transmission of COVID-19 can be controlled by adopting the right control approaches. (2) The new social evacuation policy in Korea aimed to control the epidemic further, but it did not work as expected. This may be because it reduces the public’s sense of crisis. This indicates that adopting the wrong control approach may accelerate the spread of COVID-19. (3) Finally, Mexico is an example of how the natural environment can undermine our efforts to control the epidemic. These parameters are used as mediators to establish links between the strategies implemented to control COVID-19 and the increasing process of COVID-19 patient numbers. These links can be used to guide decisions on non-drug intervention strategies.

The parameters for the six examples are given in Table 3. By looking at these parameters, we can clarify the function of the parameters in characterizing the spread of COVID-19. *λ*, *k*, and *d* jointly determine the trend of the COVID-19 transmission process (we believe that non-drug intervention strategies cannot affect *μ*, so it was not studied). When *dλ-μ*>0, the COVID-19 activity case number increases, it indicates that the COVID-19 transmission remains severe; conversely, when *dλ*-*μ*<0, the COVID-19 activity case number decreases, the COVID-19 transmission is under control. It can also be found that the larger the |*dλ*-*μ*| faster the increase or decrease of COVID-19 active cases.

Then, we do two simulations, as shown in Figs 5 and 6. The purpose of these two simulations is to clarify the influence of single parameter change on transmissions. Measures to reduce *k* (e.g., the earlier discovery of infectious diseases) can immediately reduce the COVID-19 activity case number during the observation period. Moreover, this approach’s influence on the COVID-19 spread is consistent throughout the observation period. This shows the importance of early detection and early implementation of intervention strategies. Comparatively, implementing measures to reduce *λ* and *d* is relatively ineffective in limiting the spread of the epidemic at the beginning of the observation period, but this effect becomes greater as the observation period proceeds. This suggests that the long-term impact of reducing the rate and batch of infection on transmission is more significant and increases with time after a widespread and rapid transmission of an epidemic. Therefore, it is more reasonable and economical to adopt different plans to reduce *k*, *λ*, and *d* in the face of different infectious disease transmission situations.

The simulation results of the proposed model were compared with the actual reported data to show that this model can well capture the dynamics of the increasing process of the infectious diseases patient number. In addition, the experimental results of this paper are compared with those of some effective studies based on the SIR model [65–67]. It can be intuitively found that the model proposed in this paper can better capture the transmission process of COVID-19. This is due to the fact that the study was improved in two ways. On the one hand, the models based on SIR assume that the observation area is closed and the total population in the area is constant. This assumption does not align with reality in the current context of frequent international and interregional communication. Moein, Nickaeen [66] also noted this issue. On the other hand, patients in the same state of the SIR model were considered as a whole for the calculation. This assumption is also not fully in line with reality. These two issues are fully considered in the construction of the model in this paper so that better experimental results are achieved.

## 5. Conclusions & future research directions

### 5.1. Conclusions

In this study, we propose a new Markov model for infectious diseases that requires the measurement of only four parameters, which has an infinite state space because of the unnecessary setting of maxima. To solve this model, we develop a simulation method for heterogeneous infections. Numerical experiments were conducted to analyze six regions with high transmission of infectious diseases using COVID-19 as an example. These analyses reveal that the model proposed in this paper can effectively capture the spread process of infectious diseases which are similar to COVID-19 and assess the effectiveness of the strategies being implemented to limit infectious diseases. The approach proposed in this paper extends research in infectious disease modeling and provides new ideas for predicting infectious disease transmission and designing effective intervention strategies.

Our study can provide the following recommendations for controlling and limiting COVID-19 transmission:

1. Outbreaks should be detected early, and widespread spread should be prevented. If COVID-19 does not spread widely in the area, the number of patients is not uniform in different areas. In this case, subregional control (intercity travel restrictions) is an effective approach. This is an effective way to prevent the wider spread of the virus; When the virus has been widely distributed in the observation area, subregional control is not an effective control method, so other more effective control measures should be selected.
2. According to the two simulations in Figs 5 and 6, we believe that control *λ* and *d* can continuously play a role in virus transmission, but the role of *λ* and *d* may be different in different regions. How it works is influenced by the region’s climate, population, the epidemic’s spread, and so on. In the actual work of controlling the spread of COVID-19, the role of *λ* and *d* in the region can be judged according to the actual data of different regions, and appropriate control strategies can be adopted based on this.
3. Existing studies have found several ways to reduce the spread of COVID-19, but now countries generally implement multiple control measures simultaneously. We cannot judge from the actual situation how a single control strategy affects *k*, *λ*, and *d*. Considering that COVID-19 may exist in human life for a long time, relevant institutions can clarify the role of a single control strategy through small-scale practical experiments and select appropriate methods for large-scale implementation based on the results of experimental studies.

### 5.2. Limitations and future research

This study has a few limitations, which can be addressed by future research.

First, the proposed approach has high demands on the devices for computing and storage. This is due to the fact that the model describes the infectious disease transmission process in more detail. If necessary, the calculation process can be optimized to increase the speed of the calculation.

Second, the model was constructed without considering incubation patients or asymptomatic patients. The presence of latent or asymptomatic patients can cause additional infections. Considering the incubation of patients for constructing new models is the focus of our future research.

Finally, some countries have adopted mass vaccination against COVID-19. Some vaccinated people are not infected with infectious diseases9, even if they are exposed to the virus. So, this approach will reduce the transmission of infectious diseases. It is also a good study to construct a new model of infectious disease transmission considering vaccine immunization.

## Supporting information

S1 File The derivation of the model proposed in this paper. (DOC)

S1 Table Data on COVID-19 cases of India from Nov. 1 to 20, 2020. (DOC)

S2 Table Data on COVID-19 cases of Egypt from Nov. 1 to 20, 2020. (DOC)

S3 Table Data on COVID-19 cases of Italy from Nov. 11 to 30, 2020. (DOC)

S4 Table Data on COVID-19 cases of Mexico from Nov. 13 to Dec. 2, 2020. (DOC)

S1 Fig The growth process of confirmed cases of India. (DOC)

S2 Fig The growth process of confirmed cases of Egypt. (DOC)

S3 Fig The growth process of confirmed cases of Italy. (DOC)

S4 Fig The growth process of confirmed cases of Mexico. (DOC)
