# Supplementary material for: A data-driven Markov process for infectious disease transmission
Source: PLoS One. 2023 Aug 10;18(8):e0289897. doi: 10.1371/journal.pone.0289897 (PMC10414655; doi:10.1371/journal.pone.0289897)
Supplement: S1 File — (DOC) [file pone.0289897.s001.doc]

The COVID-19 transmission model proposed in this paper is inspired by a continuous-time level-dependent Quasi-Birth-And-Death (QBD) process proposed in Li [1]. The QBD process is a special structured Markov chain whose infinitesimal generator can be generally described as follows.

$Q=\left(\begin{matrix} A_{1}^{(0)} & A_{0}^{(0)} & & & \\ A_{2}^{(0)} & A_{1}^{(0)} & A_{0}^{(0)} & & \\ & A_{2}^{(0)} & A_{1}^{(0)} & A_{0}^{(0)} & \\ & & \ddots& \ddots& \ddots\end{matrix}\right)\text{，}$ (1)

where $A_{2}^{(i)}$, $A_{1}^{(i)}$, $A_{0}^{(i)}$ are matrices and the cells in these matrices indicate the rate of state transitions. $A_{2}^{(i)}$ gives the rate that the state at level $i$ transfers to the previous level, $A_{1}^{(i)}$ gives the rate that the state at level $i$ transfers to its own state, and $A_{0}^{(i)}$ gives the rate that the state at level $i$ transfers to the next level. The state of level $i$ does not transfer to the state of other levels.

**Theorem 1** Let $\mathbf{\alpha}=\left(\alpha_{1},\alpha_{2},\alpha_{3},\ldots\right)$ be the initial probability vector of the Markov chain. Let $\mathbf{P}(t)$ be the probability vector of Markov chain at time t. From the Chapman-Kolmogorow equations, it is easy to see that

$\mathbf{P}\left(t\right)=\mathbf{\alpha}\text{ }\exp\left\{Qt\right\}\text{， }t\geq0.$ (2)

Based on the above, a new COVID-19 transmission model developed in this paper. Let $k$ be the number of cases at the time the COVID-19 outbreak was revealed. The process of virus infection before the outbreak was revealed was defined as the level 0 of the COVID-19 transmission process. The set of states at level 0 is shown as

$\text{level }0:\text{ }\{1,2,3,\ldots,k-1\},$ (3)

and the first state of level 1 is $k$. Let each patient infect $d$ patients. Then the first state of level 2 is $k(d+1)$. The set of states at level 1 and level 2 is shown as

$\text{level 1}:\text{ }\{k,k+1,k+2,\ldots,k(d+1)-1\},$ (4)

$\text{level 2}:\text{ }\{k(d+1),k(d+1)+1,k(d+1)+2,\ldots,k(d+1)^{2}-1\},$ (5)

Further, it is easy to see that

$\text{level }n:\text{ }\{k(d+1)^{n-1},k(d+1)^{n-1}+1,k(d+1)^{n-1}+2,\ldots,k(d+1)^{n}-1\},n\geq3.$(5)

Then, let each patient infect $d$ patients at rate $\lambda$ and be cured or self-cured at rate $\mu$. We have state $N$ transferred to state $N(d+1)$ at rate $N\lambda$ and to $N-1$ at rate $N\mu$.

$Q=\left(\begin{matrix} A_{0\text{，}0} & A_{0,1} & & & \\ A_{\text{1，}0} & A_{\text{1，1}} & A_{\text{1，2}} & & \\ & A_{\text{2，1}} & A_{\text{2，2}} & A_{\text{2，3}} & \\ & & \ddots& \ddots& \ddots\end{matrix}\right),$ (6)

where

$A_{n+1,n}=\left(\begin{matrix} & & & & k(d+1)^{n}\mu\\ & & & & \\ & & & & \\ & & & & \\ & & & & \end{matrix}\right),n\geq0$ (7)

(8)

Specifically, when $n$ = 0,

$A_{0,0}=\left(\begin{matrix} -\tau& & & & \\ \mu& -(\lambda+\mu) & & & \\ & 2\mu& -2(\lambda+\mu) & & \\ & & \ddots& \ddots& \\ & & & \left(k-1\right)\mu& -\left(k-1\right)(\lambda+\mu) \end{matrix}\right),d+1>k-1,$ (9)

$A_{0,0}=\left(\begin{matrix} -\tau& & & & & & & \\ \mu& -(\lambda+\mu) & \cdots& \lambda& & & & \\ & \ddots& \ddots& & \ddots& & & \\ & & a\mu& -a(\lambda+\mu) & \cdots& a\lambda& & \\ & & & \left(a+1\right)\mu& -\left(a+1\right)(\lambda+\mu) & & & \\ & & & & \ddots& \ddots& & \\ & & & & & \left(k-1\right)\mu& -(k-1)(\lambda+\mu) & \end{matrix}\right),a(d+1)\leq k-1\leq(a+1)(d+1),$ (10)

$A_{n,n+1}=\left(\begin{matrix} k(d+1)^{n-1}(\lambda+\mu) & \overbrace{\cdots}^{d} & & & & \\ & & \left[k(d+1)^{n-1}+1\right](\lambda+\mu) & \cdots& & \\ & & & \ddots& & \\ & & & & \left[k(d+1)^{n}-1\right](\lambda+\mu) & \cdots\end{matrix}\right),n\geq1$ (11)

Specifically, when $n$ = 0,

$A_{0,1}=\left(\begin{matrix} \tau& \cdots& & & & & \\ & & \lambda& \cdots& & & \\ & & & & \ddots& & \\ & & & & & (k-1)\lambda& \cdots\end{matrix}\right),d+1\geq k$ (12)

$A_{0,1}=\left(\begin{matrix} \tau& \cdots& & & & \\ \vdots& \ddots& & & & \\ & & (a+1)\lambda& & & \\ & & & \ddots& & \\ & & & & (k-1)\lambda& \vdots\end{matrix}\right),a(d+1)<k<(a+1)(d+1)$(13)

Let $\mathbf{\omega}=\left(0, 0, \ldots, 0, 1, 0, 0, \ldots\right)$ be the initial probability vector of the COVID-19 transmission model. where, 1 is at the (k+1)st position of vector $\mathbf{\omega}$, while all the other cells are zero. Let $\mathbf{P}(t)$ be the probability vector of the COVID-19 transmission model at time t.

**Lemma 1** From the Chapman-Kolmogorow equations, it is easy to see that

$\mathbf{P}\left(t\right)=\mathbf{\omega}\text{ }\exp\left\{Qt\right\}\text{， }t\geq0,$ (14)

Then, the average number of COVID-19 patients at time $t$ ($E\left[N\left(t\right)\right]$) is given by

$\begin{matrix} E\left[N\left(t\right)\right]=\mathbf{P}\left(t\right)\times N(t) \\ \text{ =}\mathbf{P}\left(t\right)\times\left(0,1,2,3,\ldots\right)^{T} \\ \text{ }=\mathbf{\omega} \exp\left\{Qt\right\}\times\left(0,1,2,3,\ldots\right)^{T}, t\geq0, \end{matrix}$ (15)

**Reference:**

1. Li Q-L. Constructive computation in stochastic models with applications: the RG-factorizations: Springer Science & Business Media; 2011.
