# Supplementary material for: A data-driven Markov process for infectious disease transmission
Source: PLoS One. 2023 Aug 10;18(8):e0289897. doi: 10.1371/journal.pone.0289897 (PMC10414655; doi:10.1371/journal.pone.0289897)
Supplement: S1 Table — (DOC) [file pone.0289897.s002.doc]

S1 Table. Data on COVID-19 cases of India from Nov. 1 to 20, 2020.

| **Date** | **Confirmed cases** | **Daily confirmed cases** | **Disappearing cases** | **Daily disappearing cases** | **Active cases** |
| --- | --- | --- | --- | --- | --- |
| 1-Nov | 8229313 | 45231 | 7667405 | 53781 | 561908 |
| 2-Nov | 8267623 | 38310 | 7726218 | 58813 | 541405 |
| 3-Nov | 8313876 | 46253 | 7780089 | 53871 | 533787 |
| 4-Nov | 8364086 | 50210 | 7836124 | 56035 | 527962 |
| 5-Nov | 8411724 | 47638 | 7890951 | 54827 | 520773 |
| 6-Nov | 8462080 | 50356 | 7945448 | 54497 | 516632 |
| 7-Nov | 8507754 | 45674 | 7995089 | 49641 | 512665 |
| 8-Nov | 8553657 | 45903 | 8043984 | 48895 | 509673 |
| 9-Nov | 8591730 | 38073 | 8086465 | 42481 | 505265 |
| 10-Nov | 8636011 | 44281 | 8141354 | 54889 | 494657 |
| 11-Nov | 8683916 | 47905 | 8194622 | 53268 | 489294 |
| 12-Nov | 8728795 | 44879 | 8244248 | 49626 | 484547 |
| 13-Nov | 8773479 | 44684 | 8292760 | 48512 | 480719 |
| 14-Nov | 8814579 | 41100 | 8335363 | 42603 | 479216 |
| 15-Nov | 8845127 | 30548 | 8379649 | 44286 | 465478 |
| 16-Nov | 8874290 | 29163 | 8420889 | 41240 | 453401 |
| 17-Nov | 8912907 | 38617 | 8466102 | 45213 | 446805 |
| 18-Nov | 8958483 | 45576 | 8515180 | 49078 | 443303 |
| 19-Nov | 9004365 | 45882 | 8560571 | 45391 | 443794 |
| 20-Nov | 9050597 | 46232 | 8610850 | 50279 | 439747 |

* Data source: https://github.com/CSSEGISandData/COVID-19.
