# Supplementary material for: A data-driven Markov process for infectious disease transmission
Source: PLoS One. 2023 Aug 10;18(8):e0289897. doi: 10.1371/journal.pone.0289897 (PMC10414655; doi:10.1371/journal.pone.0289897)
Supplement: S2 Table — (DOC) [file pone.0289897.s003.doc]

S3 Table. Data on COVID-19 cases of Egypt from Nov. 1 to 20, 2020

| **Date** | **Confirmed cases** | **Daily confirmed cases** | **Disappearing cases** | **Daily disappearing cases** | **Active cases** |
| --- | --- | --- | --- | --- | --- |
| 1-Nov | 107736 | 181 | 105833 | 115 | 1903 |
| 2-Nov | 107925 | 189 | 105943 | 110 | 1982 |
| 3-Nov | 108122 | 197 | 106070 | 127 | 2052 |
| 4-Nov | 108329 | 207 | 106192 | 122 | 2137 |
| 5-Nov | 108530 | 201 | 106335 | 143 | 2195 |
| 6-Nov | 108754 | 224 | 106449 | 114 | 2305 |
| 7-Nov | 108962 | 208 | 106594 | 145 | 2368 |
| 8-Nov | 109201 | 239 | 106710 | 116 | 2491 |
| 9-Nov | 109422 | 221 | 106819 | 109 | 2603 |
| 10-Nov | 109654 | 232 | 106934 | 115 | 2720 |
| 11-Nov | 109881 | 227 | 107067 | 133 | 2814 |
| 12-Nov | 110095 | 214 | 107177 | 110 | 2918 |
| 13-Nov | 110319 | 224 | 107276 | 99 | 3043 |
| 14-Nov | 110547 | 228 | 107388 | 112 | 3159 |
| 15-Nov | 110767 | 220 | 107499 | 111 | 3268 |
| 16-Nov | 111009 | 242 | 107644 | 145 | 3365 |
| 17-Nov | 111284 | 275 | 107769 | 125 | 3515 |
| 18-Nov | 111613 | 329 | 107916 | 147 | 3697 |
| 19-Nov | 111955 | 342 | 108072 | 156 | 3883 |
| 20-Nov | 112318 | 363 | 108206 | 134 | 4112 |

* Data source: https://github.com/CSSEGISandData/COVID-19.
