# Supplementary material for: A data-driven Markov process for infectious disease transmission
Source: PLoS One. 2023 Aug 10;18(8):e0289897. doi: 10.1371/journal.pone.0289897 (PMC10414655; doi:10.1371/journal.pone.0289897)
Supplement: S3 Table — (DOC) [file pone.0289897.s004.doc]

S5 Table. Data on COVID-19 cases of Italy from Nov. 11 to 30, 2020

| **Date** | **Confirmed cases** | **Daily confirmed cases** | **Disappearing cases** | **Daily disappearing cases** | **Active cases** |
| --- | --- | --- | --- | --- | --- |
| 11-Nov | 1028424 | 32961 | 415066 | 9713 | 613358 |
| 12-Nov | 1066401 | 37977 | 431347 | 16281 | 635054 |
| 13-Nov | 1107303 | 40902 | 443377 | 12030 | 663926 |
| 14-Nov | 1144552 | 37249 | 456117 | 12740 | 688435 |
| 15-Nov | 1178529 | 33977 | 466039 | 9922 | 712490 |
| 16-Nov | 1205881 | 27352 | 488097 | 22058 | 717784 |
| 17-Nov | 1238072 | 32191 | 504262 | 16165 | 733810 |
| 18-Nov | 1272352 | 34280 | 529184 | 24922 | 743168 |
| 19-Nov | 1308528 | 36176 | 546857 | 17673 | 761671 |
| 20-Nov | 1345767 | 37239 | 568591 | 21734 | 777176 |
| 21-Nov | 1380531 | 34764 | 588785 | 20194 | 791746 |
| 22-Nov | 1408868 | 28337 | 602921 | 14136 | 805947 |
| 23-Nov | 1431795 | 22927 | 634946 | 32025 | 796849 |
| 24-Nov | 1455022 | 23227 | 656636 | 21690 | 798386 |
| 25-Nov | 1480874 | 25852 | 689177 | 32541 | 791697 |
| 26-Nov | 1509875 | 29001 | 714030 | 24853 | 795845 |
| 27-Nov | 1538217 | 28342 | 750324 | 36294 | 787893 |
| 28-Nov | 1564532 | 26315 | 775224 | 24900 | 789308 |
| 29-Nov | 1585178 | 20646 | 789407 | 14183 | 795771 |
| 30-Nov | 1601554 | 16376 | 813083 | 23676 | 788471 |

* Data source: https://github.com/CSSEGISandData/COVID-19.
