# Supplementary material for: A data-driven Markov process for infectious disease transmission
Source: PLoS One. 2023 Aug 10;18(8):e0289897. doi: 10.1371/journal.pone.0289897 (PMC10414655; doi:10.1371/journal.pone.0289897)
Supplement: S4 Table — (DOC) [file pone.0289897.s005.doc]

S7 Table. Data on COVID-19 cases of Mexico from Nov. 13 to Dec. 2, 2020

| **Date** | **Confirmed cases** | **Daily confirmed cases** | **Disappearing cases** | **Daily disappearing cases** | **Active cases** |
| --- | --- | --- | --- | --- | --- |
| 13-Nov | 997393 | 5558 | 838964 | 5711 | 158429 |
| 14-Nov | 1003253 | 5860 | 843620 | 4656 | 159633 |
| 15-Nov | 1006522 | 3269 | 848732 | 5112 | 157790 |
| 16-Nov | 1009396 | 2874 | 853061 | 4329 | 156335 |
| 17-Nov | 1011153 | 1757 | 856977 | 3916 | 154176 |
| 18-Nov | 1015071 | 3918 | 861553 | 4576 | 153518 |
| 19-Nov | 1019543 | 4472 | 866465 | 4912 | 153078 |
| 20-Nov | 1025969 | 6426 | 871551 | 5086 | 154418 |
| 21-Nov | 1032688 | 6719 | 872101 | 550 | 160587 |
| 22-Nov | 1041875 | 9187 | 880780 | 8679 | 161095 |
| 23-Nov | 1049358 | 7483 | 886619 | 5839 | 162739 |
| 24-Nov | 1060152 | 10794 | 894255 | 7636 | 165897 |
| 25-Nov | 1070487 | 10335 | 901634 | 7379 | 168853 |
| 26-Nov | 1078594 | 8107 | 907823 | 6189 | 170771 |
| 27-Nov | 1089998 | 11404 | 908431 | 608 | 181567 |
| 28-Nov | 1101403 | 11405 | 909040 | 609 | 192363 |
| 29-Nov | 1107071 | 5668 | 924052 | 15012 | 183019 |
| 30-Nov | 1113543 | 6472 | 929526 | 5474 | 184017 |
| 1-Dec | 1122362 | 8819 | 936582 | 7056 | 185780 |
| 2-Dec | 1133613 | 11251 | 944132 | 7550 | 189481 |

* Data source: https://github.com/CSSEGISandData/COVID-19.
