# Supplementary material for: A data-driven Markov process for infectious disease transmission
Source: PLoS One. 2023 Aug 10;18(8):e0289897. doi: 10.1371/journal.pone.0289897 (PMC10414655; doi:10.1371/journal.pone.0289897)
Supplement: S2 Fig — (DOC) [file pone.0289897.s007.doc]

**
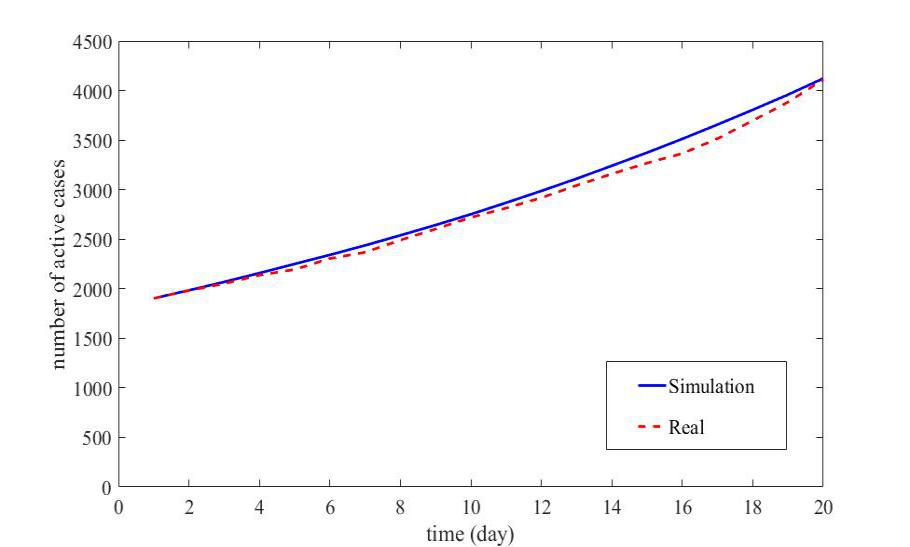
**

S4 Figure. The growth process of confirmed cases of Egypt. According to the data of Egypt from Nov. 1 to 20, 2020, the four parameters within this period are obtained: The average infection rate ($\lambda$) is 0.085434063 and the average disappearing rate ($\mu$) is 0.045978143. $d_{1}$=1, $d_{2}$=2, and the weights ($r$) of $d_{1}$ and $d_{2}$ are 0.946 and 0.054, respectively. The initial number of active cases ($k$) at the beginning of our observation period (Nov. 1) is determined as 1903.
