# Supplementary material for: A data-driven Markov process for infectious disease transmission
Source: PLoS One. 2023 Aug 10;18(8):e0289897. doi: 10.1371/journal.pone.0289897 (PMC10414655; doi:10.1371/journal.pone.0289897)
Supplement: S3 Fig — (DOC) [file pone.0289897.s008.doc]

**
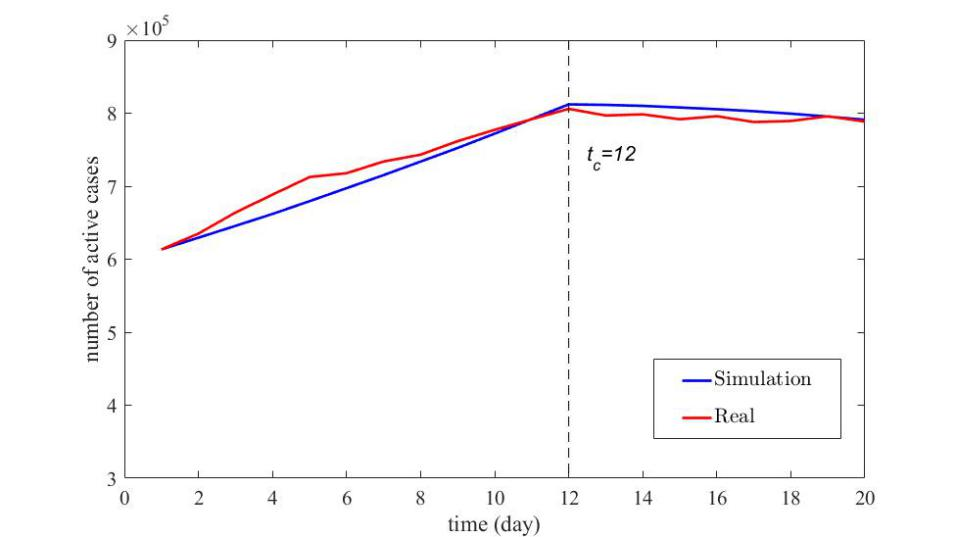
**

S6 Figure. The growth process of confirmed cases of Italy. The parameters of the observation period in Italy were as follows: $t_{c}$=12. The parameters before $t_{c}$ are as follows: The average infection rate is 0.049487386 ($\lambda$) and the average disappearing rate ($\mu$) is 0.023181119. The initial number of active cases ($k$) at the beginning of our observation period (Nov. 11) is determined as 613358. We then simulated the development of active cases in Italy during this period. In order to obtain simulated results close to the real data, we infer that the weights ($r$) of the two groups $d_{1}^{1}$=1, $d_{2}^{1}$=2 equal 0.999 and 0.001, respectively. The parameters after $t_{c}$ are as follows: The average infection rate ($\lambda$) is 0.030904889 and the average disappearing rate ($\mu$) is 0.031409968. the weights ($r$) of the two groups $d_{1}^{2}$=0, $d_{2}^{2}$=1 equal 0.550 and 0.450, respectively.
